# Artificial Mitochondria Transfer: Current Challenges, Advances, and Future Applications

**DOI:** 10.1155/2017/7610414

**Published:** 2017-07-02

**Authors:** Andrés Caicedo, Pedro M. Aponte, Francisco Cabrera, Carmen Hidalgo, Maroun Khoury

**Affiliations:** ^1^Colegio de Ciencias de la Salud, Escuela de Medicina, Universidad San Francisco de Quito (USFQ), 170901 Quito, Ecuador; ^2^Colegio de Ciencias Biológicas y Ambientales, Instituto de Microbiología, Universidad San Francisco de Quito (USFQ), 170901 Quito, Ecuador; ^3^Mito-Act Research Consortium, Quito, Ecuador; ^4^Colegio de Ciencias Biológicas y Ambientales, Universidad San Francisco de Quito (USFQ), 170901 Quito, Ecuador; ^5^Colegio de Ciencias de la Salud, Escuela de Medicina Veterinaria, Universidad San Francisco de Quito (USFQ), 170901 Quito, Ecuador; ^6^Institute for Regenerative Medicine and Biotherapy (IRMB), INSERM U1183, 2 Montpellier University, Montpellier, France; ^7^Laboratory of Nano-Regenerative Medicine, Faculty of Medicine, Universidad de Los Andes, Santiago, Chile; ^8^Consorcio Regenero, Chilean Consortium for Regenerative Medicine, Santiago, Chile; ^9^Cells for Cells, Santiago, Chile

## Abstract

The objective of this review is to outline existing artificial mitochondria transfer techniques and to describe the future steps necessary to develop new therapeutic applications in medicine. Inspired by the symbiotic origin of mitochondria and by the cell's capacity to transfer these organelles to damaged neighbors, many researchers have developed procedures to artificially transfer mitochondria from one cell to another. The techniques currently in use today range from simple coincubations of isolated mitochondria and recipient cells to the use of physical approaches to induce integration. These methods mimic natural mitochondria transfer. In order to use mitochondrial transfer in medicine, we must answer key questions about how to replicate aspects of natural transport processes to improve current artificial transfer methods. Another priority is to determine the optimum quantity and cell/tissue source of the mitochondria in order to induce cell reprogramming or tissue repair, in both in vitro and in vivo applications. Additionally, it is important that the field explores how artificial mitochondria transfer techniques can be used to treat different diseases and how to navigate the ethical issues in such procedures. Without a doubt, mitochondria are more than mere cell power plants, as we continue to discover their potential to be used in medicine.

## 1. Introduction

Mitochondria are cell organelles descended from an alphaproteobacterial endosymbiont [1] and play a fundamental role in growth, differentiation, and survival beyond sustaining the energetics of the cell [2, 3]. Diseases, tissue damage, and aging challenge the cell and its mitochondria, thereby affecting their integrity, function, and homeostasis [4, 5]. Cells naturally have the capacity to exchange intracellular material and especially mitochondria through different processes such as cell-to-cell contact, microvesicles, nanotubular structures, and other mechanisms [6–8]. Clark and Shay pioneered the artificial mitochondria transfer (AMT), which involved transferring mitochondria with antibiotic-resistant genes into sensitive cells, thereby enabling them to survive in a selective medium [9] and opening this new field of research. Since the work of Clark and Shay, the process of artificial transfer has and continues to mimic aspects of naturally occurring cell transport, especially in the mechanisms cells naturally use to rescue other damaged cells. The AMT restores and increases respiration and proliferation and completes other cellular processes [5, 10–16].

This review will consider key advances necessary to improve the current knowledge about the artificial transfer of mitochondria and how these techniques could be used therapeutically. We will provide an overview of the features of the mitochondrial structure that are important in maintaining its integrity throughout artificial transfer [13, 14]. Next, we will discuss how a cell naturally protects the mitochondria during their transport by using intercellular bridges or microvesicles and the effects of the transferred mitochondria in the receiver cell [6, 17, 18]. The in vivo artificial transfer of mitochondria was carried out at the same time as many in vitro assays [5, 7, 12, 13, 16, 19]. These approaches will be covered in the third section. For example, those assays performed by McCully in 2009 [16] and recently by Huang et al. in 2016 [19] raised questions about the best source of mitochondria, what kinds of stress during their transfer could affect mitochondrial function or prevent their arrival to the target tissue, among other questions. The key to developing new lines of research in this field is determining the diseases in which AMT could be effective as well as the potential advantages of such therapeutic treatments over others. Taking this into account, it is essential that we further study the effectiveness of different donor sources of mitochondria in repairing recipient cells and determine how such findings can help to establish ethical guidelines that will facilitate future safety research and enable the development of new medical applications of AMT. Without a doubt, more advances are needed to better understand and improve AMT and lay the foundation for its safe use in treating mitochondrial damage and related diseases.

## 2. Structural and Functional Characteristics of Mitochondria for a Successful Artificial Transfer

The mitochondrion is an organelle present in most of eukaryotic cells; it is in charge of ATP synthesis via oxidative phosphorylation (OX-PHOS), calcium metabolism, and the control of the apoptotic intrinsic pathway, among other functions. At present, the mitochondrion is recognized as an endosymbiotic organism, whose noneukaryotic origin could facilitate its ability to be transferred from one cell to another. It has a double protective membrane and partial transcriptional independence from the nucleus, thereby making the mitochondria an item which can naturally be exchanged by microvesicles and nanotubes between cells [20–22]. Given that there is no cellular protection when performing AMT, it is important to conserve mitochondrial integrity after isolation when exposed to an extracellular environment. The isolation procedure and stressors present outside the cell or organism like temperature change and surrounding media would greatly modified the structural stability, function, and potential effects of the mitochondria in the receiver cell [23]. In this section, we will focus on key biological aspects that should be taken into consideration when the AMT to other cells is sought.

The mitochondria evolved from a prokaryotic organism, and when it colonized the first protoeukaryotic cell, it developed a system of close communication with the nucleus by exchanging its own mtDNA sequences with it [24, 25]. It is estimated that mitochondria need almost 2000 proteins to work properly, but in many species, mtDNA encodes barely 63 proteins or less [26, 27] and most of these proteins are synthesized in the cytoplasm by means of ribosomes encoded in the nucleus and not by those of the mitochondria, thereby making them partially independent [28]. The interaction between nuclear and mitochondrial genes is essential for the organelle transcription, translation of proteins, and respiration [29]. Considering this close relationship, the compatibility between the mitochondria of one cell or species interacting with the nucleus of another could potentially affect their crosstalk, thereby inhibiting cell respiration and function [29–32]. These specific differences in the nuclear and mitochondria genome between cells or species could cause incompatibility if the auto, allo, and xenogenic AMT is pursued [13].

The mitochondria's small size as well as its capacity to change its shape and length allows it to be transported by subcellular transporting mechanisms such as tunneling nanotubes (TNTs) and microvesicles (MVs) [33, 34]. Its diameter varies between 0.5 and 1.0 *μ*m, and its length shows great variability, from 0.5 to 10 *μ*m. Although its shape is defined as rounded or elongated, mitochondria can be very pleomorphic, or in other words, they may exhibit great morphological variations. Some mitochondria could be fused and interconnected in networks, in contrast to the classic bean shape that appears in most illustrations [35, 36]. This organelle is characterized by a double lipoprotein membrane, each of which are about 7 nm thick. The outer mitochondrial membrane is smooth, biochemically identical to the membranes of eukaryotic cells, and rich in cholesterol (possibly contributing to the cell capacity to internalize this organelle when it is free in external medium) [11].

Guaranteeing the integrity of the outer and inner membranes during any process of AMT between one cell to another is key to protecting this organelle's function and the effects on the receiver cell after transfer. The outer mitochondria membrane (OMM) serves as a barrier and a platform to exchange products between that cytoplasm and the intermembrane space [37, 38]. The OMM also protects the cell from any harmful product, like free radicals from the active metabolic processes carried out by the mitochondria [37, 39]. OMM permeabilization can be induced by toxins, *gamma* and/or UV irradiation, hypoxia, and growth factor deprivation causing irreparable mitochondria DNA (mtDNA) damage. These factors can lead to the activation of proapoptotic multidomain Bcl-2 proteins, such as Bax or Bak [40–43]. A permeabilized or fragile OMM would not be effectively internalized after AMT by the receiver cell or even could activate apoptotic processes instead of repairing or increasing cellular functions [11]. Further studies should be completed in order to fully understand the interactions between the OMM and the receiver cell membrane and to understand the process of uptake.

The inner mitochondrial membrane (IMM) is chemically similar to bacterial cell membranes and rich in cardiolipin, a phospholipid made of 4 fatty acids that decreases this membrane's permeability to protons. The IMM's lack of proton permeability is essential because it allows the existence of differential concentrations between the mitochondrial compartments (intermembranous space and mitochondrial matrix). The IMM is composed of the inner boundary membrane (IBM) and cristae membrane (CM), where the IBM is opposed to the OMM and the CMs are extended protrusions of the IBM inside the matrix [44]. The CM's shape is created by multiple folds in the membrane. This allows more IMM to be packed into the organelle and thereby provides scaffolding for the electron transport chain complexes and ATP synthase which represent 80% of the protein mass of the inner membrane [45, 46]. The disruption of the IMM architecture could result in the alteration of the cristae dynamics in the mitochondria, consequentially affecting its capacity to fuse with other mitochondria and to produce ATP [37, 47, 48]. One of the therapeutic possibilities of AMT is enabling the exchange of mtDNA from exogenous healthy mitochondria to damaged receiver mitochondria thereby contributing to the ATP production in which maintaining the integrity of IMM could favor the process.

Mitochondrial fitness is essential to maintain the integrity and functioning of the cell. Many reactions take place inside the mitochondria and are the consequence of its good condition, among fatty acids *β*-oxidation, Kreb's cycle, urea cycle, heme biosynthesis, and part of the steroid, cardiolipin, and ubiquinone biosynthesis pathways. Genetic variations in mitochondria and the presence of deleterious mutations in their DNA can alter their structure, function, and integrity. Many crucial aspects of their physiology are still not fully understood which are necessary to understand how physiological changes or stressors, like subproducts of the electron transport chain (i.e., reactive oxygen species (ROS)) and others induced by the environment (contamination or age), can damage components of the mitochondria. In order to develop more efficient mechanisms and succeed the AMT, we must find ways to maintain their structural integrity during AMT, guaranteeing that the outer and inner membrane structures will be conserved and also that the mitochondria does not lose its function during the transfer, thus assuring the beneficial effects of the procedure [9, 14]. Previous work about the AMT evaluates mitochondrial function by fluorescent probes and electron microscopy being a key aspect of the transfer procedure [14, 16, 49, 50]. Picard et al. observed in 2011 that the isolation procedure of the mitochondria from cells or tissues induces the fragmentation of the organelle, modulates the permeability of the transition pore sensitivity to calcium, alters the respiration rates of oxygen consumption, and increases the mitochondrial stress-producing free radicals [23]. There is still no information about the absolute or relative number of damaged versus healthy mitochondria during the AMT process. Obtaining this information could contribute to a better evaluation and comparison of the different AMT methods discussed in this review.

Cells and mitochondria change during the process of differentiation. It has been described that stem cell mitochondria are in a dormant and immature state: they are small and favor anaerobic metabolism. Through the process of differentiation and loss of their pluripotency, mitochondria proliferate and the quantity of DNA, the rate of respiration, and the generation of ATP synthase increase. These changes cause the mitochondria to develop an elongated morphology and swollen cristae. Its matrix also becomes more dense, being relocated to a wider extent in the cells [51–54]. It has not been studied whether the isolated mitochondria show variations on their effects on the recipient cells depending on the differentiation states as mitochondria show strong differences on their structure and metabolic profiles. Questions like whether the cristae distribution change, ROS produced during the transfer, need to be answered and incorporated to the isolation and transfer protocols.

In the next section, we will describe key aspects of natural intercellular mitochondria transfer, a cellular function which protects other cells from damage or stress [7, 8, 33, 55]. During transport, mitochondria are enclosed and secured by membranes, thus protecting them from external damage. In order to achieve successful artificial transfer, these mechanisms will need to be recreated in order to protect the mitochondria.

## 3. Natural Intercellular Mitochondrial Transfer

To date, several groups have reported the horizontal transfer of mitochondria in different cell types in vitro and in vivo, describing a new cellular property [7, 8, 21, 56, 57]. Most of the work about mitochondrial delivery from one cell to another deals with the rescue of damaged cells by healthy ones, such mesenchymal stem cells (hMSCs) [8, 56, 58]. Additionally, other studies have linked this transfer process to MSCs' enhanced immune response to macrophages; this is just one example of the diverse effects this mechanism has on cells involved in the transfer [33]. Recently, this process was also observed occurring between astrocytes and neurons during focal cerebral ischemia [21]. Interestingly enough, in such cases, mitochondria from the retinal ganglion cell are transferred to astrocytes of the optic nerve head to be broken down and recycled [57]. From the first description of the transfer of intracellular material between cells in 2004 by Rustom et al. [6], the work of Spees et al. in 2006 [7] to the in vivo assays performed by Islam et al. in 2012 [8] and Jackson et al. in 2016 [33], most studies show that MSCs are the best cells to transfer mitochondria. Considering the potential benefits of natural mitochondria transfer, there is great urgency to better comprehend, facilitate, and artificially replicate this process.

**Figure 1:**
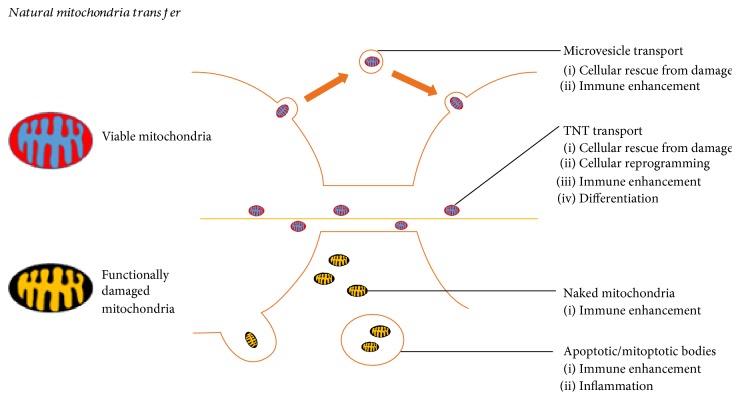
Natural mitochondria transfer. Viable and nonfunctional mitochondria can be shared by the cell inducing different cellular responses from cellular rescue to promoting inflammation. The first transfer mechanism is the microvesicle transport of mitochondria, it has been observed specially in MSCs in which the secreted microvesicles carrying mitochondria, once internalized by the recipient cells, induce its rescue from cellular damage and enhance the phagocytic properties of immune cells [182, 183]. The second way of transfer is by TNTs; many cells share the ability to produce them and transport mitochondria with proven effects in the rescue from cellular damage, metabolic reprogramming, and immune enhancement and it was also associated with its differentiation [65, 184]. During cellular stress, defective mitochondria can be released without being covered like in apoptotic or mitoapoptotic bodies and being naked promoting the immune response and inflammation [17, 77, 185].

The transport of mitochondria from one cell to another is part of the dialogue necessary to the development and maintenance of homeostasis in multicellular organisms (Figure 1) [59]. Mitochondria can travel from one cell to another by intercellular structures such as tunneling nanotubes (TNTs) and secreted cellular bodies, such as microvesicles [5, 20, 33, 60]. In 2004, Rustom et al. described TNTs as a structure that enables cell-to-cell interaction. Since then, a number of groups have studied the cells that produce TNTs and receive mitochondria and other intracellular cargo [5, 6, 33, 61]. Other reviews in this special issue and recently published work recapitulate the details of TNT structure generation, characteristics, and mitochondria transfer [60, 62–64].

TNTs are produced by the outgrowth of filopodia-like cell membrane protrusions that connect with the target cell. The membrane from each cell extends to fuse together, thereby forming a tightly connected bridge which is independent from any substrate [22]. TNTs contain a skeleton mainly composed of F-actin and transport proteins like MIRO1 that facilitate the active transfer of cargo and mitochondria along these structures [58]. TNTs were first described in rat-cultured pheochromocytoma PC12 cells [6], and subsequent studies have shown that they connect a wide variety of cell types. These studies provide more evidence that TNTs are involved in mitochondrial transport between cells, the repair of cell damage, the activation of enhanced immune responses, and cell metabolic reprogramming [5, 8, 33, 61, 65].

The directionality of the transport of intracellular material and mitochondria through the TNTs is not fully understood. It is important to define what factors promote the donation of material and their effects on the recipient cells. Sun et al. observed that TNTs' growth is guided by the extracellular protein S100A4 and its putative receptor RAGE (receptor for advanced glycation end product). Stressed hippocampal neurons and astrocytes initiated the formation of TNTs after p53 activation. This signaling pathway triggered caspase 3, which decreased S100A4 in injured cells and caused cells with a high level of S100A4 to become receptor cells [66]. By these results, the authors proposed that damaged cells need to transfer cellular contents to healthy ones, in a process related to the spread of danger signals but no insights about mitochondrial participation were given. In contrast, Spees et al. in 2006 observed that MSCs transferred mitochondria to respiration-deficient cancer cells, but the direction of the transport or bidirectionality mode were difficult to determine due to the fact that the recipient cells were depleted of mitochondria, and it was not described whether the MSCs received any intracellular material from the cancer cells [7]. Koyanagi et al. in 2005 have shown that mitochondria are exclusively transported by TNTs from human endothelial progenitor cells to neonatal undifferentiated cardiomyocytes in a process intended to sustain their maturation [67]. Gao's team in 2016 used microfluidic channels while tracking TNTs' formation and exchange of material in coculture assays. Gao's team observed that MSCs were responsible of the TNT formation and of mitochondria transfer to cardiomyocytes, as opposite to fibroblasts (negative control of the interaction) [68]. Bidirectional transport of mitochondria is also plausible as it was observed between malignant mesothelioma cells. These cells produce more TNTs than normal mesothelioma cells, but interestingly, their proliferation was inversely correlated with TNT formation during their culture in low serum, hyperglycemic, acidic growth medium [69]. These represent just a few examples of the extensive literature about the exchange of intracellular material and mitochondria and its directionality. Yet, mitochondrial transport is not fully understood. For example, the field still needs to define the cell types that produce TNTs and deliver cargo to recipient cells [68]. The determination of the directionality of and conditions necessary for mitochondria transfer between different cells is essential to understanding the potential role of this process in helping cells exposed to stress or during the transmission of danger warning signals among cells. Another important question that still remains unanswered is the reason why MSCs have a greater propensity to form TNTs compared with other cells.

Many groups of researchers that use lung disease models have corroborated that mitochondria can be transferred to other cells in vivo. Islam et al. in 2012 reported that bone marrow-derived stem cells (BMSCs) could be used to supply healthy mitochondria to alveolar epithelial cells in a mouse model of *E. coli* LPS-induced acute lung injury [8]. The delivery of mitochondria into injured cells increased ATP levels which in turn maintained cellular bioenergetics and recovered epithelium functions. A follow-up study in lung disease models (rotenone-induced lung injury and allergen-induced asthma) contributed to the understanding of the mechanisms involved in mitochondrial transfer through nanotubes, confirmed the protective effect of mitochondrial donation, and revealed a Miro1-regulated mitochondrial movement from MSC to damaged recipient epithelial lung cells [58]. All these data corroborate that mitochondrial delivery can rescue damaging cells. Furthermore, in a mouse model of *E. coli*-induced pneumonia and acute respiratory distress syndrome (ARDS), transfer of mitochondria from MSC toward innate immune cells by TNTs enhanced macrophage bacterial phagocytosis in the harmed tissue, thus improving the process of repair [33].

Extracellular vesicles (EVs) are also involved in the transport of intracellular cargo to other cells. EVs are spheroid structures surrounded by a lipid bilayer membrane [70] and are capable of transporting proteins, lipids, carbohydrates, metabolites, small RNAs [71], and mtDNA [17]. EVs are classified depending on their size and biogenesis. This classification includes exosomes (30 to 100 nm in diameter), microvesicles (100 nm to 1 *μ*m in diameter), and apoptotic bodies (1 to 2 *μ*m in diameter) [72]. Apoptotic bodies have been less studied due to their rapid elimination by phagocytic cells [70]. Lastly, exosomes and microvesicles are released by diverse cell types including, platelets, endothelial cells, and breast cancer cells [34]. Both mRNA and microRNA have been found in exosomes and could be transported to target cells [73]. Guescini et al. in 2010 also observed the delivery of mtDNA by exosomes. mtDNA can also be delivered via exosomes, as it was detected in glioblastoma cells and astrocytes [74]. The full understanding of the mechanisms of mitochondrial transfer by EVs and effects in receiver cells are still unclear.

It has been observed that the nervous system benefits from the transfer of mitochondria for different purposes. For example, the transfer of mitochondria allows cells to breakdown nonfunctional mitochondria and to transfer healthy mitochondria to stressed neurons. The process of mitochondrial transfer is not always meant to protect damaged cells but also to recycle these organelles in other cells in a process called transcellular degradation of mitochondria or transmitophagy [57]. The transmitophagy process is mediated by cellular evulsions containing mitochondria from neurons, in which these structures are embraced by astrocytes and then recycled [57]. The reason why transmitophagy takes place is still unknown, but it has been hypothesized that focal axon damage stimulates the process. Another theory posits that transporting damaged mitochondria back to the neuron soma is energetically disadvantageous and that there are specialized astrocytes to perform this task and clearance of unfunctional mitochondria [57]. Astrocytes are responsible for protecting and repairing damaged neurons through several mechanisms in which the transfer of mitochondria by extracellular MVs containing vascular endothelial growth factor (VEGF), fibroblast growth factor 2 (FGF-2), and mitochondria is vital to support cell recovery after stroke or cellular stress [21, 75]. Understanding transmitophagy and the natural transfer of mitochondria by microvesicles in the nervous system will allow us to find new therapeutic options in which this processes could mediate the recovery of neurons' homeostasis and function in degenerative diseases.

One of the multiple mechanisms by which MSCs exert their natural therapeutic effects is via EVs. In 2012, Lee et al. isolated the exosomes from mouse and human MSCs. Subsequently, they injected the MSC-derived exosomes into the murine model of hypoxic pulmonary hypertension (HPH) and observed the therapeutic effects of MSC action in the tissue [76]. The same study found that MSCs prompt depolarized mitochondria to move to the outer limits of the plasma membrane in response to a higher concentration of oxygen (21%). This movement is mediated by the arrestin domain with protein 1-mediated MVs larger than 100 nm. Finally, these MVs are secreted and fuse with macrophages, thus enhancing their oxygen consumption rate and most likely improving their therapeutic properties as well.

MSCs secrete exosomes with microRNAs, thus inhibiting the activation of the macrophages and repressing the TLR signaling. Phinney et al. in 2015 found an association between this response and a mechanism in which MSCs make macrophages more susceptible to acquiring exogenous vesicles and mitochondria [17]. In 2012, Cho et al. [56] replicated the assays performed by Spees in 2006 [7] in which he cultured MSCs with human osteosarcoma 143B cells, subsequently causing their mitochondria to become compromised or depleted [7]. Cho et al. observed that MSCs actively transferred healthy mitochondria by nanotubular structures to the 143B mitochondria-depleted cells [56]. Cho et al. treated the MSCs with rhodamine 6G in order to alter mitochondria activity but not mtDNA. The authors observed that fully functional mitochondria were needed to recover the loss of respiration of the 143B mitochondria free cells. In a crucial part of the experiment, they cocultured MSCs with cells carrying mtDNA mutations (A3243G mutation or 4977 bp deletion) and saw no recovery of function [56].

Cells tend to dispose of their mitochondria when they are unfit after exposure to stress conditions or when keeping them becomes harmful as mitochondria can produce large quantities of ROS [77]. Around 30 to 50% of the highly glycolytic HeLa cells were able to survive after their mitochondria were damaged by ejecting them through selective elimination or mitoptosis [77]. During this process, the mitochondria were degraded by their inclusion in membrane vesicles and exocytosis. The presence of degraded mitochondria and especially of mtDNA in the extracellular space has been associated with a proinflammatory response and the presence of antimitochondria antibodies such as anticardiolipin and antisarcosine dehydrogenase, which are characteristic of sepsis and associated with negative patient outcomes [77].

The mtDNA and ROS released by eosinophils have been shown to provide antimicrobial protection; they also represent a key component of the innate immune response [78]. Stimulated LPS hepatocytes and mouse embryonic fibroblasts extrude mitochondrial material through autolysosomal exocytosis, thereby activating polymorphonuclear leukocytes [79]. The release of mitochondrial contents activated inflammatory responses [79]. Lastly, intact mitochondria from necroptotic cells may play a role in hazard signaling when they are ejected from cells during tumor necrosis factor alpha (TNF *α*) induced necroptosis. These mitochondria are engulfed by macrophages and dendritic cells, resulting in the secretion of proinflammatory cytokines by macrophages and dendritic cell maturation [80].

A handful of in vivo studies have shown that mitochondria can be released either naked or encapsulated by a membrane bilayer. Nakajima et al. in 2008 used a mouse model to confirm that naked mitochondria are released into the intercellular space after an anti-Fas antibody injection. In response to this treatment, cytoplasmic vacuoles engulfed fragmented mitochondria and extruded them from apoptotic hepatocytes [81]. Likewise, activated platelets released respiratory-competent mitochondria, both as free organelles and encapsulated within the microparticles. These extracellular mitochondria mediate inflammatory responses [82]. Elucidating the mechanisms involved in mitochondrial extrusion will lead to a better comprehension of the diseases produced by dysfunctional mitochondria and inflammatory disorders.

Apparently, cells, especially MSCs, use mitochondria as a direct reprogramming agent because the mitochondria are independent from receptors or coupled proteins to induce their effects. Cytokines, miRNAs, transcription factors, and other cell components require the activation of specific signal pathways in order to induce a response of proliferation, growth, or other in cells [83, 84]. However, we can speculate that the exogenous mitochondria, once inside the cell, start to breathe and fuse with other mitochondria. These characteristics or mechanisms make the transport of mitochondria through TNTs or vesicles important to their protection and ensure their integrity and stability. We do not know if mitochondria free in circulation or inside microvesicles have the same effects or which one could better induce proliferation, cell repair, or other [5, 8, 33, 67, 68]. Another issue is if the isolating protocol of mitochondria when applied to cells or tissues could damage its function and effects in cells [85].

The quest for the most efficient method to deliver mitochondria in vitro and in vivo remains ongoing. Based on currently available literature, it appears that achieving effective AMT will require us to preserve the integrity and effectiveness of the mitochondria by protecting them within membrane structures, such as microvesicles. Since the first description of the mitochondrial transfer from MSCs to mitochondria-depleted cells [7], numerous studies have been conducted in vivo and in vitro. The results of these studies have provided more evidence to this novel field of research. Understanding these cell properties opens a new avenue for the development of therapeutic strategies like AMT to the treatment of mitochondrial-related disorders.

## 4. Artificial Mitochondria Transfer (AMT)

**Figure 2:**
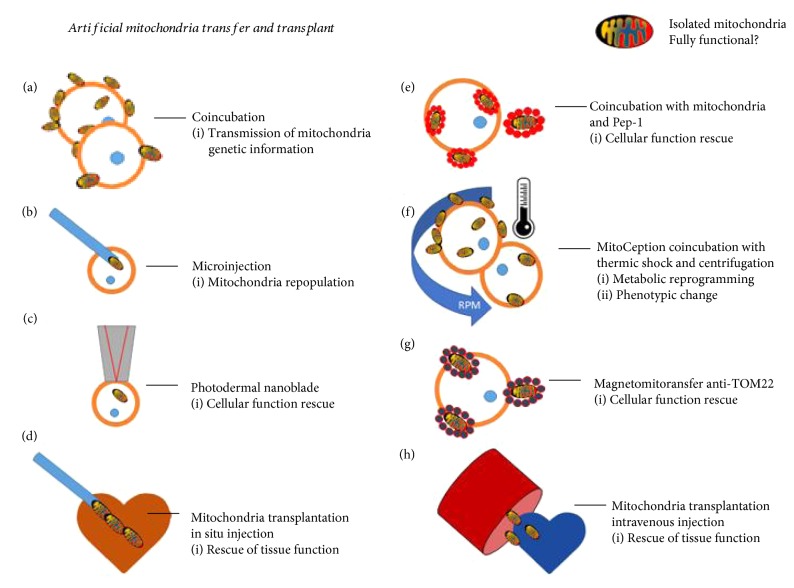
Artificial mitochondria transfer (AMT) and transplant. Different techniques emerged to mimic the natural transfer or mitochondria on its in vivo and in vitro applications. The coincubation technique was the first proposed in which the antibiotic resistance carried in the mitochondrial DNA was passed to sensitive cells [9], later after the technique was used to rescue respiratory deficient cells among other damaged cells [10, 11, 14, 63]. Microinjection of exogenous mitochondria was applied in assays to eliminate the endogenous copies of oocytes carrying mitochondrial diseases [90, 93]. The photothermal nanoblade effectively transferred isolated mitochondria inside the cell; even if they showed great effectiveness, its application is limited to small cell numbers [12]. Two different approaches were developed to facilitate the mitochondria internalization in the recipient cells, the first is by using Pep-1 and the other with magnetic beads (Magnetomitotransfer) designed to bind to TOM22 a receptor complex in the mitochondrial membrane. The MitoCeption technique uses a thermic shock and a centrifugation to improve the process of mitochondria uptake; first applied in cancer cells, this technique induces the metabolic reprogramming of these cells. The in vivo application of the mitochondria transfer applies two approaches: the first is to directly inject mitochondria to the harmed tissue and the other in the circulatory system close to the area of interest. Both of them have shown to restore tissue function but the in situ injection showed better results [16, 49, 50].

Without a doubt, the mitochondrion is the master organelle of cell energetics, fueling multiple processes like proliferation, migration, differentiation, and stress resistance [86–89]. The transfer of mitochondria between cells through nanotubes or microvesicles stimulates these processes and also protects the recipient cells from stress-related injury. Several research teams are currently working on AMT in order to understand how to promote cellular repair in this context [5, 8, 10, 55]. Since the first formal mitochondrial transfer from one xenogeneic cell to another was completed by Clark and Shay in 1982 through coincubation [9, 19], this rapidly growing field has developed new AMT methods in order to observe its effects in recipient cell types and imagine new possible applications (Figure 2).

In 1982, Clark and Shay developed “Mitochondrial Transformation,” the very first technique to transfer mitochondria from one cell to another [9]. In their model, they were able to transform around 30,000 recipient cells in just one procedure, making it a highly efficient method. They used the antibiotics chloramphenicol (CAP) and efrapeptin (EF), which inhibit the mitochondria's protein synthesis and ATPase function in order to kill sensitive mammalian cells. Cells resistant to CAP have mutations in their mtDNA located in one region of the mitochondrial large subunit rRNA gene [9]. They observed that the transfer of mitochondria from CAP and EF-resistant fibroblasts increased the survival of the recipient cells, which were sensitive to these antibiotics. Interestingly, they observed that when the mitochondria of sensitive cells are transferred to new cells, they did not confer resistance to the recipient cells. This provides evidence that a higher concentration of mitochondria in and of itself is not sufficient to protect cells from CAP or EF; rather, mitochondria can only survive by having the genes for antibiotic resistance. It was also apparent that mitochondria from murine fibroblasts which were resistant to CAP and EF did not increase the survival of sensitive human cells. This indicates that mixing endogenous and transferred mitochondria across different species could potentially be restricted. A crucial observation of this article is that the failure of AMT into murine cells by simple coincubation suggests that this process is not equally efficient among different cell types and that some cells may be more receptive than others [9]. Clark and Shay's observations and questions regarding the mechanism of mitochondrial transfer have opened up the path for further advances in the field.

In 1988, King and Attardi then developed the first AMT technique using invasive instruments; they injected exogenous mitochondria isolated from CAP resistant cells into sensitive human cells [90]. Their method was less efficient than Clark and Shay's coincubation protocol because the technique limited the number of cells that could be transformed in each procedure and caused harm to the recipient cell. However, this study demonstrated that the injection of just one mitochondrion could very quickly repopulate a cell depleted of its endogenous mitochondria in just six to ten weeks. Additional techniques to perform AMT involving nanoblades and other invasive instruments have been developed, but all of them are less efficient than coincubation [12, 91].

Mitochondria carrying genetic mutations can cause diseases that can be transmitted to offspring through the oocyte [92]. To prevent and treat such diseases, experiments have utilized a variety of different AMT approaches, from microinjecting healthy mitochondria into oocytes [93] to transferring the nucleus of an unfertilized, mutation-carrying oocyte to a healthy enucleated ovule. Using King and Attardi's AMT technique, Pinkert et al. used microinjection to transfer mitochondria isolated from the livers of *Mus spretus* to fertilized oocytes taken from *Mus musculus* [93]. After 4.5 days in culture, Pinkert et al. detected xenogeneic mitochondria DNA sequences in the recipient cells, thereby demonstrating that xenogeneic mitochondria from closely related species are able to survive in recipient oocytes for at least a limited time [93]. In 2007, Yi's team observed that zygotes that had received mitochondria transferred from the livers of young mice developed better through the blastocyst stage, as compared to old zygotes that did not undergo AMT [94]. In 2010, Takeda et al. used the mitochondria from bovine fibroblasts cultured in 10% and 0.5% serum [95]. Interestingly, the oocytes that received mitochondria isolated from cells at 0.5% serum showed a lower rate of development [95]. Recently, the transfer of mitochondria by microinjection has been replaced by enucleating the ovule of a healthy donor and adding the zygote nuclear material from a carrier of mitochondrial mutations in a pronuclear state and in metaphase II [96]. Although these techniques have been successful [97], they are still ethically controversial due to the amount of germ cells sacrificed in the procedure; these issues will be discussed in a later section of this review.

Clark and Shay's assay [9, 14] coincubates isolated mitochondria with the recipient cell, a technique which can be easily applied to many different types of cells. This procedure provides an opportunity to study the behavior and effects of artificially transferred exogenous mitochondria inside recipient cells. Despite other successes using this technique, in 2005, AMT unexpectedly failed when Spees et al. coincubated mitochondria isolated from hMSCs with human lung carcinoma recipient cells (A549) [7]. Before the procedure, A549 cells were pretreated with ethidium bromide in order to deplete their mtDNA and to make the cells unable to perform aerobic respiration, just as King and Attardi had done before [90]. When Prockop's group cocultured the depleted A549 cells with hMSCs, they observed the natural transfer of mitochondria between them. This transfer appeared to rescue respiration of the dysfunctional A549 cells. After their coincubation assay failed, they hypothesized that the transfer of mitochondria is mediated by active mechanisms such as the formation of nanotubular structures like TNTs or vesicles which transport these organelles to the interior of recipient cells [6]. Unknown details about temperature changes during the mitochondria isolation may have been instrumental to understanding the lack of passive transfer. Two years later, in a xenogeneic model, Weissig successfully transferred isolated mitochondria from mouse livers into human cancer cells, MDA-MB-231 and MCF-7. Katrangi et al. tested this technique in four models, using each type of cancer cell with and without mitochondria depletion by ethidium bromide [13]. The success of each of these models can be attributed to the methodology they used to isolate the mitochondria. The key of their methodology was maintaining the mitochondria at 4°C at all times in order to preserve their structure and function. They also maintained the cells in normal culture medium with uridine and pyruvate. The success of this protocol provides insights into the specific conditions that recipient cells may need in order to successfully internalize exogenous mitochondria and to prevent changes in cellular function and metabolism due to exposure to temperature variability. Another possibility is that some cells may be more receptive to accepting mitochondria. For instance, cancer cell lines such as MDA-MB-231 naturally incorporate more material from their surroundings in a process described as entosis or cell cannibalism [98].

Supporting Weissig's work (2007), the same year Yoon et al. observed that mitochondria from different species have the ability to fuse together. Their study did not use the mitochondria transfer technique, but instead they fused the cells with polyethylene glycol (PEG). They also labeled human and mice mitochondria differently (mtGFP and mtDsRed, resp.) and observed a mix of the two types of mitochondria 45 min after adding the PGE and fusion of the mitochondria of all hybrids at 4 h. The fusion of both human and mice mitochondria apparently occurs because of the homology of the sequence between the proteins responsible for this process, the mitofusins proteins (Mfn1 and Mfn2). Mfn1 and Mfn2 share a 90.7% and 94.8% homology between humans and mice. The formation of the mitofusin homodimers between the membranes of both types of mitochondria initiates the tethering and the fusion of the inner membrane [99]. However, even when mitochondria from both species fused, Yoon et al. were not able to create long-term cybrids from the mouse-human fusion, although they were able to achieve it from mouse-mouse fusions. This can be explained by the accumulation of differences between species, especially within the nuclear-coded mitochondrial genes and the dialogue between mitochondria and nuclei; it appears that long-term crosstalk between the nucleus and the xenomitochondria cannot be established [99, 100]. Yoon's work on the compatibility of xenomitochondria with human cells demonstrates that successful mitochondrial transfer may only be possible between cells and/or tissues of the same species.

In 2012, Elliott et al. coincubated mitochondria isolated from immortalized breast epithelial cells with their malignant counterparts MCF7, MDA-MB-231, and ADR-Res. They observed a decrease in their proliferative potential and a higher sensitivity to chemotherapeutic drugs such as doxorubicin, abraxane, and carboplatin [101]. Interestingly, they observed that only isolated mitochondria from the immortalized breast epithelial cells were able to enter the breast cancer cells, but not the original immortalized breast epithelial cells [101]. It was not further discussed in the article whether the characteristics of the immortalized cells' mitochondria are different from those of normal epithelial cells or if such differences could affect the process of their integration into the immortalized and cancer cells. To sustain the transfer of isolated mitochondria into recipient cells by coincubation, Kitani et al. demonstrated that this process can be performed autogeneically and xenogeneically. They documented the results of the transfer through real-time PCR and fluorescent imaging [11]. Nevertheless, although they obtained functional cells with integrated xenomitochondria, Yoon was unable to show the permanence of the exogenous mtDNA for longer than two weeks [99].

Assays to optimize transfers involving the use of chemical compounds and physical methods have been performed since 1988. In 2013, Liu and colleagues conjugated isolated mitochondria with penetrating peptides to foster their internalization. They used Pep-1, a cell-penetrating peptide that was originally developed to induce pores in the membrane to facilitate the delivery of molecules like oligonucleotides into the cell. The authors adapted Pep-1 to conjugate it with isolated mitochondria of human osteosarcoma 143B cells [15]. The mix of Pep-1 and the isolated mitochondria promoted their internalization by fibroblasts involved in a model of the mitochondrial disease myoclonic epilepsy with ragged red fiber (MERRF) syndrome. They observed that the Pep-1-mediated transfer was more successful in facilitating the internalization of mitochondria than mitochondria alone [102]. Unfortunately, the authors did not describe the efficacy of or the rationale behind conjugating Pep-1 and mitochondria. They also did not describe the unexpected lack of internalization of the exogenous mitochondria not conjugated with Pep-1. Interestingly, in 2016, Liu's team used the Pep-1-conjugated allogeneic and xenogeneic mitochondria in an in vivo assay of a Parkinson disease model (PDM) [103]. They observed an increase of neuron survival and movement recovery in the animals of the experimental group as compared with the control. Yet, questions arise from Lui's technique, such as whether the Pep-1 peptide acts as a protective agent from environmental damage or whether it acts as an internalizing agent that facilitates the transfer of mitochondria to the affected tissues. Finally, defining the optimal quantity of the mitochondria administered to the PDM in rats will be important in studying the possibility of applying this technique to treat neurodegenerative disorders or any other disease.

In 2015, our group standardized the transfer mechanism of isolated mitochondria to cultured cells (MDA-MB-231, human breast cancer cells), adding two extra steps to the coincubation procedure: centrifugation and a thermic shock. We named the protocol *MitoCeption*. Our technique allowed the constant and reproducible increase of mitochondrial uptake by the recipient cells proportionally to the material added. Following these observations, we could prove an equivalent increase of respiration and ATP production in accordance with the supplementation of mitochondria. We witnessed a functional change in the mitocepted cancer cells: their proliferative and invasive potential increased. Interestingly, we also observed that a cancer cell cannot constantly incorporate mitochondria without harming their functional properties; the proliferation and invasive capacities of the cells diminished after increasing their mitochondria concentrations. Our work showed that it is useless to constantly improve the mitochondria transfer mechanism if there is a functional threshold of the internalized mitochondria. It is clear that this technique cannot be used for the in vivo transfer of mitochondria to organs or tissues, but cells can be mitocepted before being introduced into in a living organism with the purpose of reprogramming or repairing its metabolism and function [10].

The transfer of mitochondria by coincubation seems to depend on the viability and metabolic activity of the donor mitochondria and particularly on the integrity of the outer membrane, as this is the first structure organelle to interact with the receiver cell. Kesner et al. in 2016 further studied the coincubation transfer mechanism using isolated mitochondria from Hela cells and transferring them to other cancerous cell lines, healthy fibroblasts, and cells carrying mitochondrial mutations. They noted that the uptake was fast, with recipient cells uptaking the exogenous mitochondria just 10 minutes after the coincubation began. Kesner et al. also established that the mitochondrial transfer is principally mediated by macropinocytosis [14], which is a regulated form of endocytosis mainly involved in the uptake of molecules, nutrients, and other materials from the extracellular space [104, 105]. Furthermore, they found that perturbing the outer membrane of mitochondria with digitonin or other mitochondria-damaging molecules inhibits the uptake process. Their key observations provided insights about how mitochondria integrity is important to the success of the transferring process. They also importantly noted that membrane characteristics of the recipient cells may play a major role in the macropinocytosis of mitochondria.

Wu et al. developed a photothermal nanoblade to deliver cargo including mitochondria to the interior of mammalian cells, bypassing cell fusion and endocytosis [12]. Progressive resistance BTK-143 osteosarcoma and MDA-MB-453 p0 cells lacking mtDNA were treated with the photodermal nanoblade in order to deliver HEK293T-expressing mitochondria labeled with DsRed. The goal of this procedure was to rescue the metabolic function of the receiving cells. This technique effectively transferred exogenous mitochondria, but due to the technical expertise and equipment required to carry out this procedure, the results obtained in the experiment are difficult to replicate. Furthermore, as the authors mention, the transfer of mitochondria by the photothermal nanoblade is low throughput, meaning that the technique must be adapted in order to achieve the same efficiency as coincubation or MitoCeption techniques. Later the same year, Macheiner and colleagues [91] developed the use of anti-TOM22 magnetic beads to improve the purity of mitochondria isolates. They used a magnet to transfer the mitochondria coupled with the magnetic beads into host cells, naming this technique Magnetomitotransfer [91]. TOM22 is a multisubunit translocase embedded in the outer membrane of the mitochondria [106]. Coupling the mitochondria with anti-TOM22 beads increased the quantity of viable mitochondria able to be transferred. However, because the beads can also bind with nonfunctional mitochondria fragments, they may also inadvertently transfer them into recipient cells. According to the authors, a greater ratio of transfer was achieved using this technique after one to three days of culture as compared to passive transfer. By the same token, Kesner had already observed that the process of internalization of mitochondria via coincubation can occur in as little as 10 minutes [14]. This observation could put into question the need of the magnetic beads to accelerate the process. The authors also did not show whether the fusion of exogenous and endogenous mitochondria is affected in some way by the anti-TOM22 beads. Fusion is an essential process for the exchange of mtDNA between mitochondria; therefore, this method may not actually be effective if fusion is not facilitated by the anti-TOM22 beads. As they mentioned, further studies are needed to learn about the toxicity and changes in cell physiology after magnetic mitochondrial transfer, especially those associated with respiration and metabolic reprogramming. Both the photothermal nanoblades and Magnetomitotransfer are still limited in terms of the number of cells that they can reach, the damage they may cause, and the greater technical challenges involved in executing each procedure [10].

In 2009, McCully et al. proved that mitochondria can be used in vivo to repair damaged tissues [16]. Because ischemic damage affects the mitochondria in tissues, these authors hypothesized that the replacement of affected mitochondria with healthy ones would significantly improve the postischemic recovery. They induced ischemia in the heart of rabbits by occluding the left coronary artery using the Langendorff perfusion allowing to test the contractile strength and heart rate. Then, they injected either a vehicle, vehicle with mitochondria, or mitochondria alone which had been thawed after an overnight period at −20°C in the presence of the vehicle. These were injected directly into the ischemic zone just before reperfusion. Interestingly, they observed that the infarcted area was reduced and the functional recovery increased after injecting mitochondria combined with the vehicle. This was not observed in the tissue only injected with mitochondria isolates, meaning that mitochondria must be active in order to serve therapeutic functions, as described in a previous work in vitro [13]. The authors used healthy heart tissue from rabbits as a supply of mitochondria, which limited the impact of the study. This strategy has many translational limitations. The use of other sources, like unharmed tissue from the same donor or other nonvital tissue from other rabbits, would have provided greater evidence for further applications.

Later in 2013, Masuzawa et al. added further assays to sustain the efficacy of the transfer of mitochondria in the ischemic heart model in rabbits [49]. This time, they isolated mitochondria from the pectoral muscle of the same rabbit used for the ischemic shock (autologous transfer). After a follow-up of 28 days, the authors observed that the autologous transplantation of mitochondria was not proarrhythmic: infarct marker levels decreased and the generation of precursor metabolites for energy and cellular respiration increased. Interestingly, they also found that mitochondria were internalized by the cardiomyocytes 2 hours after transplantation, with cardioprotective effects after 28 days. The proposal of a mechanism of mitochondria transfer and their internalization in vitro based on macropinocytosis by Kitani et al. and Kesner et al. [11, 14] was not conclusive. In their latest contribution, McCully's team (2015) observed that the transfer of mitochondria and their in vivo internalization was mediated by actin-dependent endocytosis and not by macropinocytosis. The authors used different inhibitors to prevent the internalization of mitochondria. They used cytochalasin D to inhibit actin polymerization, methyl-*β*-cyclodextrin (M*β*CD) to stop endocytosis, and nocadozole to block tunneling nanotubes. They observed that the use of (M*β*CD) greatly inhibited the uptake process, inferring that internalization is mainly mediated by actin-dependent endocytosis [50]. Further assays need to be developed in vivo to fully understand the process of internalization related to AMT, possible heterogeneity across different tissues, and the effects of the transfer of mitochondria to harmed tissue. Such assays may also help to illuminate ways to improve AMT and address its limitations.

After McCully's experiments in 2013, Lin et al. applied the same procedure to replace damaged mitochondria with healthy ones to mitigate symptoms in the rat model of hepatic ischemia-reperfusion [107]. Both McCully and I-Rue Lai observed that the introduction of mitochondria to diseased ischemic tissue decreased damage and oxidative stress and improved recovery. Despite this success, these experiments were not able to make any conclusions about three factors which may be key to the success of mitochondria therapies: extract concentration, mitochondria viability, and organ-to-mitochondria ratio. With respect to the first factor, neither experiment demonstrated a dose response related to the concentration of isolated mitochondria introduced to the damaged tissue. McCully used 9.7 × 10^6^ ± 1.5 × 10^6^/ml of mitochondria isolated from healthy hearts in injections of 0.1 ml, eight times into the affected zone of the ischemic hearts [49]. In his study, I-Rue Lai used a concentration of 7.7 × 10^6^ ± 1.5 × 10^6^/ml of mitochondria isolated from healthy livers in one injection of 0.1 ml into the subcapsular region of the spleen poles. Although each concentration yielded therapeutic benefits, neither was established as the optimal injection concentration. Additionally, each study verified the viability of the isolated mitochondria before injecting them into the given tissue using fluorescent probes dependent on membrane potential, including CMTMRos [107], JC1, and respirometry [49]. However, it remains unclear how the mitochondria's state at the moment of injection affects the success of the therapy. We cannot assume that the mitochondria's optimal injection state is simply indicated by activity, because activity is not related to the coupling of the electron transport chain or ROS production, which can damage the mitochondria and the cell [108]. Additionally, these studies did not consider the impact of organ-to-mitochondria ratio, although this factor may have important implications in the successes of mitochondria therapy. McCully and I-Rue Lai's studies are highly important because they demonstrate that AMT in vivo can have therapeutic benefits to the damaged renal and cardiac tissues; perhaps even more impactfully, they have also opened new lines of investigation to help us understand how to optimize these procedures for clinical applications.

In 2014, Sun et al. transferred mitochondria to the damaged lung tissue of adult male rats affected by acute respiratory distress syndrome (ARDS) [109]. In this experiment, Hon-Yap Yip and colleagues transferred mitochondria, melatonin, and mitochondria in combination with melatonin to diseased lung tissue. Melatonin was used because it is a known anti-inflammatory molecule which is protective against lung injury disease [110]. Islam et al.'s previous experiment in which they observed the transfer of mitochondria from bone marrow-derived stromal cells (BMSCs) to harmed lung tissue served as the foundation for Hon-Yap Yip's work [8]. Islam et al. demonstrated that BMSC mitochondria transferred to the affected alveolar epithelia resulted in an increase of cell bioenergetics and had additional protective effects [8]. Taking into consideration the work of Islam et al. [8], Masuzawa et al. [49], and Lin et al. [107], Sun et al. [109] completed onetime intravenous injections of two different mitochondria concentrations (not coupled with melatonin) 750 *μ*g and 1500 *μ*g diluted in IBc buffer in rats 6 hours after inducing ARDS. They did not specify the total volume they injected. In other assays, the authors injected mitochondria in combination with melatonin and melatonin alone. In both cases, the melatonin was injected in a concentration of 50 mg/kg, 6 and 24 hours after ARDS was induced. Sun et al. [109] observed that the treatment with just mitochondria and mitochondria plus melatonin decreased DNA damage, ROS generation, apoptosis, and the quantity of albumin in bronchoalveolar lavage (BAL), an indicator of capillary leakage in proteins and proinflammatory cytokines such as MMP-9, TNF-*α*, and NF-*κ*B, [109]. The fact that this study requires a great quantity of mitochondria makes it less likely that it could be successfully applied in larger organisms. However, this transfer method could be better used in localized injections, as put into practice by McCully et al. in their studies [16, 49].

Huang et al. transferred mitochondria isolated from the kidney of young hamsters to rats that had suffered a cerebral stroke induced by middle cerebral artery occlusion (MCAO) [19, 109]. The authors discovered that the administration of the xenogenic mitochondria had protective effects and were associated with a faster recovery of motor performance in the rats. They injected 75 *μ*g of mitochondria diluted in 10 *μ*l of SEH solution (0.25 M sucrose, 0.5 mM ethylene glycol tetraacetic acid (EGTA), and 3 mM N-(2-hydroxyethyl) piperazine-N¢-ethanesulfonic acid (HEPES), pH 7.2) into the ischemic stratum and infused 750 *μ*g in 100 *μ*l into the femoral artery. Interestingly, the authors showed that the direct in situ injection of the isolated mitochondria were more effective in rescuing motor activity than the mitochondria injected through the artery. They also showed that the exogenous mitochondria have a low percentage of internalization in the harmed neural cells, but even so, the low internalization rate seems to be sufficient to exert their protective properties.

The application of the AMT in vivo should be further developed because all investigations to date have only used one or two mitochondria doses [16, 19, 107, 109]; the similarity of this aspect of research protocols limits our understanding of the effects and therapeutic applications of mitochondria. Hon-Yap Yip [109] injected isolated mitochondria intravenously and Hong Lin-Sun [19] injected mitochondria intra-arterially; they compared the limitations of the therapeutic effects of these methods with those of the in situ infusion. It will be important to further study if the regenerative properties of mitochondria injected into the circulatory system are comparable with direct infusion into the damaged site. Some possible limitations of the systemic infusion may include the loss of integrity of the mitochondrial membrane, the delay in arriving to the damaged site, and the possibility that others cell in the circulatory system endocytose the mitochondria, thus restricting the quantity of mitochondria that ultimately arrive to the harmed tissue.

Two trends in the study and application of the AMT between cells have emerged in the last 30 years. In 1982, Clark and Shay created the mitochondrial transformation technique, based on the simple coincubation of isolated mitochondria and cultured cells [9]. In 2009, McCully et al. innovated the direct in vivo approach [16]. These techniques established important questions to guide the future development of AMT. For example, they revealed that mitochondrial integration may not occur equally between cells and that it may be possible for cells with different membrane properties or tissue organization to be similarly transformed. Additionally, they brought into question how isolation techniques affect the mitochondria's functioning and integrity, and if these changes could influence the transfer itself. Lastly, they raised questions about how the genetic patrimony of donor mitochondria could influence the effects of the transferred mitochondria in the host [9]. These questions have been addressed by authors like Spees et al. [7], Katrangi et al. [13], Kitani et al. [11], Kesner et al. [14], Wu et al. [12], and Caicedo et al. [10]; however, work is still necessary in order to be able to apply AMT in clinical settings.

Many relevant questions in the field remain to be answered. For example, it is still unknown how mitochondria from different cells are able to improve or decrease cellular processes. Similarly, the field must also determine whether transferred mitochondria are able to fuse with endogenous ones and communicate correctly with the nucleus, which is essential to their long-term effectiveness. Caicedo et al. previously observed that the transfer of mitochondria from MSCs to cancer cells by MitoCeption in concentrations higher than 1.25 *μ*g (measured in protein) was deleterious for cell proliferation and that even higher concentrations (2.5 *μ*g>) were restrictive for invasion [10]. Recently, Kitani et al. [11] and Kesner et al. [14] observed that the coincubation of isolated mitochondria and cultured cells was sufficient to prompt the cells to internalize the mitochondria, thereby resulting in the repair of cellular function. However, more research is necessary to understand if additional procedures like thermic shock, centrifugation [10], cell penetration by peptides [15] with mitochondria-conjugated beads [91], or introduction by nanoblades [12] can optimize the AMT and induce the desired effects in the recipient cells.

To develop AMT procedures that are easily replicable and effective, it is important to define a mitochondria-isolating procedure standard that allows scientists to obtain pure mitochondria and analyze the effects of transfer. The isolation of mitochondria is based on the conditions and speed of centrifugation, the concentration of the sucrose solution, and some other factors which, when managed incorrectly, can contaminate the mitochondria concentrate and therefore negatively impact the therapeutic effectiveness of the AMT procedure. Contamination can happen because of the structural closeness and functional connections of the mitochondria with other organelles [85]. For example, mitochondria closely interact with the endoplasmic reticulum (ER), together forming the mitochondria-associated membranes (MAMs) which play a crucial role in calcium homeostasis, regulation of lipid metabolism, and autophagy [111, 112]. Due to the tight contact between the MAMs, contamination with ER may be common in most AMT protocols. Similarly, in other cell structures, mitochondria isolation may be contaminated with other organelles with which the mitochondria interact, including the nucleus, lysosomes, and peroxisomes [111, 113–116]. Clark and Shay [9], Katrangi et al. [13], Kitani et al. [11], Kesner et al. [14], Sun et al. [109], and Huang et al. [19] used the classic sucrose gradient with differential centrifugation, and then, Elliot et al. [101], Chang et al. [102], Macheiner et al. [91], and Lin et al. [107] used experimental kits to guarantee the purity and viability of mitochondria for downstream applications. McCully et al. did not clearly define the type of protocol used for mitochondria isolation for his in vivo procedures [16, 49, 50], but in his latest review [117], he suggested two protocols by Gostimskaya and Galkin [118] and Claude [119] which can rapidly isolate mitochondria. In 2006, Spees et al. briefly mentioned a mitochondria isolation protocol that involves differential centrifugation; however, the article may not have mentioned important details of this protocol because the experiment did not yield any successful internalization of mitochondria in incubation with the recipient cells [7]. It is possible that this protocol unexpectedly failed because of contamination with MAMs, which may influence the internalization of isolated mitochondria, although no work to date has addressed this potential problem.

Mitochondrial tissue specificity or differentiation state should be taken into account when choosing the donor mitochondria. Mitochondria differ in their shape, size, energy production, and metabolic processes among cell types and states of differentiation [120, 121]. During cell proliferation, the mitochondria modifies its dynamics, segregates from others, and fuses with other mitochondria in a process mediated by the expression of Mfn1 and Mfn2 or dynamin-related protein 1 (DRP1) in the OM [122]. Interestingly, the growth factor erv1-like (Gfer) plays an important role in regulating DRP1 in embryonic stem cells (ESCs). When Gfer is not present, DRP1 is highly expressed driving mitochondrial network fragmentation and a decrease of pluripotency in ESCs [123, 124]. Isolating mitochondria from a cell in a specific state like proliferation could prime the mitochondria and influence their impact in recipient cells' mitochondria networks or even their capacity to fuse with endogenous mitochondria. Most of the studies regarding the transfer procedure were performed using mitochondria from differentiated cells like fibroblasts, liver cells, MSCs, and others [7, 9, 13, 90, 93]. Testing different cell states will be important to understand how exogenous mitochondria interact with the endogenous organelles, how the cell's phenotype changes after transfer, and if metabolic reprogramming is possible.

AMT has shown promising results in healing damaged or stressed cells in vitro and in vivo. Understanding their mechanisms of action inside the cell will allow us to explore and mix AMT with other techniques to repair dysfunctional mitochondria. The use of AMT could potentially repair endogenous and damaged mitochondria by introducing healthy copies to recipient cells and inducing a state of heteroplasmy. In heteroplasmy, altered or pathogenic mtDNA exists together with healthy or wild-type mtDNA [125]. A cell eliminates damaged mitochondria carrying altered mtDNA by mitophagy, a key process in maintaining the mitochondria pool quality [126]. Mitochondria pass through fission in which unhealthy copies carrying altered mtDNA, those with low membrane potential, and those with excessive ROS are eliminated mainly by the PTEN-induced kinase 1 (PINK1) and Parkin pathway [127]. In recent years, the field has gained interest in mitophagy because of its implications in maintaining cell viability and stemness [125, 126, 128–130]. The inhibition of rapamycin (mTOR) kinase activity activates mitophagy, thereby enhancing the selection against dysfunctional mitochondria. However, but healthy mitochondria must also be present to induce this process [126]. Using mitophagy inducers could improve mitochondrial function in mitochondrial diseases or other conditions in which the mitochondria metabolism may be altered [131]. Understanding how AMT may induce heteroplasmy in cells carrying mitochondria mutations and encourage the clearance of unhealthy mitochondria copies in order to support the quality control of mitochondria by mitophagy may reveal new therapeutic possibilities [126].

More studies are needed to understand the possible applications and challenges of AMT. Solid preclinical assays must be designed in order to select the best mitochondria donor cells to treat a specific disease. With this approach, tuning the delivery methods of AMT will further facilitate the reconceptualization of mitochondria not only as the powerhouse of the cell but also as active therapeutic agents.

## 5. Mitochondrial Diseases and Their Potential Treatment by AMT

The pathophysiology of mitochondrial diseases is complex considering that they can be caused by mitochondrial or nuclear genes involved in the correct biogenesis and function of this organelle. At present, medical approaches for the treatment of mitochondrial disease are only palliative. For this reason, mitochondrial transfer techniques could potentially play a curative role in the care of individuals at risk for or those already suffering from mitochondrial disease caused by mutations in their DNA. Primary mitochondria diseases (PMDs) are caused by mitochondrial mutations that are inherited and transmitted by one's maternal lineage. The transmission of these mutations to successive generations causes most of the known mitochondrial disorders such as Leigh syndrome. This syndrome can affect mtDNA and nuclear DNA (nuDNA), which are considered mitochondria-associated genes [132]. Mutations in the mtDNA can occur during life, and the expression of the disease depends on the quantity of mitochondria that are damaged in comparison with healthy copies in the cell. These diseases are secondary mitochondria diseases [47]. Once an individual's cells cross a certain threshold of damaged mitochondria, the disease will manifest [133]. It is in these cases that the artificial transfer of healthy mitochondria to damaged cells or tissue may help to treat the disease.

Although usually considered rare, mitochondrial diseases appear to be more common than ever thought. Recent work establishes population frequencies of at least 1 : 5000 [134, 135]. The first links between mtDNA alterations and mitochondrial disease were established in the year 1988 [136–138], and today, more than 260 disease-causing mutations and 120 mitochondrial genome rearrangements have been classified [139]. Furthermore, carriers of asymptomatic mtDNA mutations are estimated to be 1 : 200 [134], a frequency 25 times higher than that of people actually suffering from mitochondrial disease. Because healthy carriers of genes that cause mitochondrial diseases likely do not know that they have these mutations, there is an increased risk of them passing on their potentially deleterious genes to their children.

Mitochondrial disorders are extremely difficult to diagnose because they appear with signs and symptoms common in many other non-mitochondrial-related diseases [140]. Therefore, they should be considered as syndromes. Clinical manifestations may affect any system in the body, but since mitochondria act as power stations, the most affected tissues or organs are those that manage and consume a great deal of energy. Accordingly, most common mitochondrial disorders affect the nervous system (including the sensory organs) and the musculoskeletal system. Because the reproductive system also has organs that require a lot of energy, it can also be affected by mitochondrial disease. However, such diseases have not received as much research attention as mitochondrial diseases of other systems, perhaps because they are not life-threatening. The severity of mitochondrial diseases and their negative impact on patients' quality of life emphasize the need for innovative management of these disorders.

With new therapeutic techniques, the manipulation of mitochondrial biology can allow mothers carrying mutations in their mtDNA to have healthy offspring. Used for the first time in humans in 2016, this set of technique is called mitochondria replacement (MRTs) and gives rise to “three-parent babies” [97, 141]. MRT techniques eliminate the majority of mutant mitochondria in the female germ line during very early developmental periods before the baby is even born, thereby preventing mitochondria-related morbidity entirely. One method, pronuclear transfer, transplants the nucleus from a zygote carrying mutant mitochondria to a donor-derived enucleated oocyte with healthy mitochondria [96]. The offspring receives its nuclear genome from the sperm and the nucleus from the original oocyte, which are transplanted into the donor-derived enucleated oocyte which contains cytoplasm and healthy mitochondria. Despite a few successful applications of MRT, these highly invasive techniques can cause difficulties in fertilization because of the high levels of manipulation involved. For example, maternal spindle transfer (MST) transplants the microtubular spindle system with all chromosomes attached (before pronucleus formation) into a healthy oocyte that has had its own spindle fibers removed during the same developmental stage; this technique has proven to be very effective in monkeys [142] but fertilization issues have been observed in some human spindle-transferred oocytes [143]. This technique requires that the embryo be pierced with micropipettes, which not only ruptures the cell membrane, but probably also disrupts cytoplasmic organelles and the cytoskeleton. This invasive procedure is required in all MRT techniques involving nuclear material transfer [143, 144]. Moreover, oocytes must undergo additional manipulation during the necessary ablation of the *zona pellucida* [142, 144]. During spindle transfer, several chemicals including the cancer drugs cytochalasin B and nocodazole are also applied to the cells involved, possibly damaging genetic material in the cells [142, 144]. Additionally, the use of MRTs is ethically controversial because embryos have to be destroyed during the procedure [145]. AMT offers a less invasive alternative to allow mothers with mitochondrial mutations to have healthy children, especially in cases in which there are only palliative treatments available for the given mitochondrial disease [146].

In the future, AMT could be used in conjunction with induced pluripotent stem cells (iPSCs) to model mitochondrial diseases or to generate healthy cells to be reintroduced into the patient. It is thought that diseased somatic cells could be used to generate iPSCs cells, which would then be forced to differentiate into lineage restricted adult stem cells of the specific diseased tissues. Once obtained, these cells would be inoculated with healthy mitochondria by AMT and then injected into the diseased tissue. This combinatory technique may have applications in diseases like mitochondrial retinopathy, which currently has no known curative treatments [147, 148]. It may be possible to combine AMT with currently available techniques used to derive specific retinal progenitors in order to introduce healthy mitochondria copies into diseased cells, thereby repairing the damaged mitochondrial pool and curing the disease [149].

Patients with skeletal muscular syndromes caused by mtDNA damage may also benefit from AMT. One therapeutic technique that may be able to treat such diseases involves the systemic injection of mitochondria to allow them to arrive to the target tissue. However, because the injection could become diluted in the circulatory system, a sufficient number of mitochondria probably would not reach the muscle tissue in order to heal it. The Magnetomitotransfer could offer an alternative to better guide mitochondria into the target tissue by using magnets [91]. Yet another option may be to inject a greater concentration of healthy mitochondria into small local arteries feeding specific muscles or directly into the muscle mass. This approach has been attempted in the heart, a muscle which suffers from mitochondrial damage after ischemic episodes during myocardial infarction [16, 117]. McCully et al. directly injected mitochondria into the infarcted areas and subsequently discovered that the procedure was beneficial to the recovery of the cardiac tissue [16].

The theoretical AMT procedures outlined here may have great therapeutic potential in a range of applications, from curing mitochondrial retinopathies to treating muscular skeletal syndromes [150]. However, in order to unlock the therapeutic potential of AMT, we must address a number of ethical concerns and technical challenges to safely use this techniques in humans. Significant preclinical experimentation must be performed before adapting AMT for clinical trials.

## 6. Ethical Issues

The UK has become the first country in the world to formally approve the use of mitochondrial donation, both MST and PTN [151]. This regulation was created in October 2015, under the licensing and regulation of the UK Human Fertilization and Embryology Authority (HFEA) [152]. This medical and legal advance gave families with serious mitochondrial diseases a range of possibilities to allow them to have their own genetically healthy children. In 2016, MRTs were deemed ethical by a panel of U.S. experts, provided that the procedures adhered to certain guidelines. The panel further recommended that MRT only be used to produce male babies, thereby avoiding the transmission of the surrogate donor mitochondria to future generations [153].

Prior to the approval of the UK statutory instrument, there were several round table discussions involving the public which focused on the scientific and ethical implications of MRT. These well-scrutinized debates have engendered both widespread support and significant dismay. Ultimately, the common ground that helped pass this regulation was the understanding that mitochondrial donation can prevent a child from inheriting metabolic disease, thereby offering parents with mitochondrial disease an opportunity to have healthy children. As demonstrated in this case, good regulation helps science to advance, avoid setbacks, and, ultimately, reach patients within a reasonable time frame.

On the other hand, some constituents voiced great disagreement following this debate before and even after the final approval of MRT. The responsible regulatory agencies are now obligated to develop a robust case-by-case licensing protocol which takes into account the technical challenges and ethical complexities of this procedure. This is a crucial component of regulation to avoid setbacks, to facilitate the further development of the MRT, and to continue to provide families affected by mitochondrial disease a way to ensure the health of their babies. In practical terms, for a clinic to be able to carry out mitochondrial donation, it will need to follow a two-stage licensing process: first, it will need to apply for a license and then seek additional authorization to initiate the treatment in a particular case. Having recently concluded this lengthy legalization process, the first babies conceived using MRT following this protocol are expected to be born this year in the UK.

Many experts have recommended that families that use mitochondrial donation should be encouraged but not obligated to take part in long-term follow-up studies in order to monitor any possible effects on children conceived through this technique and on future generations. This postprocedure follow-up has been deemed crucial to ensuring that the development of this technique keeps pace with ethical advancements and the evolving sociopolitical climate of the UK and the wider world. However, by the same token, there are potential pitfalls looming on the horizon. For example, “medical tourism” from countries that lack the technology or the legal approval for such procedures may attract patients to the UK. This may significantly limit clinical follow-up of children conceived with MRT and the eventual identification of any related safety issues [154].

Most of the arguments against MRT are either scientific or ethical in nature. One question that dominates this debate is about the classification of MRT as a medical procedure. Should MRT be considered more similar to egg/sperm donation or tissue/organ donation? Given that mitochondrial donation involves the transfer of genetic but not nuclear material, this has led to uncertainty as to whether it should be regulated as egg or as tissue donation [155]. Many studies have concluded that the genes that contribute to personal characteristics and traits come solely from nuclear DNA [155–157]. In other words, traits arise from a child's mother and father, not the mitochondrial donor. Although interactions between mtDNA and cellular entities including nuclear DNA do exist, there is no evidence that nuclear DNA can be altered through epigenetic or translocation mechanisms. Considering the limited genetic contribution of mitochondrial donors, MRT is more likely to be classified as a procedure similar to tissue donation. The only confirmed traits that could arise from the donor mtDNA are related to energy production; these traits are considered minor in their overall impact on the organism. For example, variations in the mitochondrial genome have been associated with subtle differences in energy metabolism, such as the ability to cope at high altitudes.

Other studies consider the contribution of mtDNA to bioenergetics [158] to be highly important given that mitochondria-related metabolic processes in the brain play an essential role in neurotransmitter release and synaptic plasticity [159]. For this reason, the great metabolic demands of normal brain function make human cognition dependent upon mitochondrial function. Impaired mitochondrial function caused by mtDNA damage may render neurons more susceptible to oxidative injury [160] and thereby allow systemic or environmental factors to exert a noticeable effect on the brain. Certain allelic variants in mtDNA genes often lead to cognitive impairments, and it has even been hypothesized that “mitochondrial dementia” may exist [161]. Supporting this argument, a great deal of other evidence suggests that mitochondrial dysfunction may play a role in psychiatric disorders such as schizophrenia, bipolar disorder, and major depressive disorder. A study of mitochondrial dysfunction in a small cohort found that increased common deletions and decreased gene expression in mitochondria was associated with increased prevalence of psychiatric illnesses [162]. The role of mtDNA in human cognition and in the onset of degenerative diseases requires further clinical investigation in large cohorts.

In many countries, ethical review committees allow parents to decide if they wish to undergo the MRT technique. Although MRT has been successfully applied, there are ongoing questions about the cost benefits of this procedure, especially related to the stress and invasiveness of the technique on the potential mother [163]. An expert UK panel commissioned on evaluating the safety of MRT concluded that the techniques were “not unsafe” based on successful trials completed in mice and monkeys [142]. An important question remains as to whether additional rigorous preclinical safety testing is still required, although the technology has already been approved for use in humans. There is emerging evidence in recent literature which raises concerns regarding MRT. For example, a recent publication highlighted the gradual loss of donor mtDNA in embryonic stem cells (ES cells) derived from MRT embryos and a reversal to the maternal haplotype. The group identified a polymorphism within the conserved sequence box II region of the D-loop as a plausible cause behind the preferential replication of specific mtDNA haplotypes. In addition, they demonstrated that some haplotypes confer proliferative and growth advantages to cells [164]. Another group provides direct evidence of mtDNA involvement in cognitive functioning. In fact, the association between mtDNA and the functioning of the nervous system during neural development and synaptic activity involves mitochondrial genes. The total substitution of mtDNA modified learning, exploration, and sensory development as well as the anatomy of the brain; all of these changes persisted with age. These findings demonstrate that mitochondrial polymorphisms are not as insignificant as previously believed [165].

Additionally, new evidence has emerged that shows that even low levels of heteroplasmy introduced into human oocytes by mitochondrial carry-over during nuclear transfer often vanish. Low levels of heteroplasmy can sometimes instead result in mtDNA genotypic drift and reversion to the original genotype [166]. It is important not to overinterpret these results, as most of the corresponding experiments were performed in mice. Furthermore, this evidence should not be used as an argument to rethink the approval of MRT, but rather to be considered when performing the recommended follow-up of children born following MRT intervention [164].

It is important to mention that both PNT (pronuclear transfer) and MST were approved by the Human Fertilization and Embryology Authority based on successful studies completed with rodents and nonhuman primates. However, they may not be equally safe because only MST has been tested in large animals. When MST was tested in mature nonhuman primate oocytes (*Macaca mulatta*), it showed normal fertilization, embryo development, and production healthy offspring [142]. A point-by-point comparison between MST and PNT is detailed in Table 1 [167]. The chart details a lower carry-over of mtDNA but higher potential risk of chromosomal abnormalities for MST [168].

AMT which does not involve any kind of nuclear transfer has great potential to satisfy both ethical and safety concerns. Mitoception involves the transferring of mitochondria from a donor to a recipient cell or tissue and does not involve the passing on of nuclear material. Nevertheless, this technique requires a thorough ethical analysis before considering clinical implementation. One central issue is the origin of donor mitochondria. This requires individual analyses of autotransfer, allotransfer, and xenotransfer because each donor source may have unique ethical and biological implications. In autotransfer, the mitochondria from a tissue with a low mtDNA mutation risk could be used to treat a highly compromised organ of the same person. This donor source poses few ethical concerns but entails great biological challenges that require further complex experimentation and the use of animal models to develop. Allotransfer would use mitochondria donations from genetically close family members. Ideally, the human donor and recipient should share the same haplotype [169]. Alternatively, if no close relatives are available, haplotype matching could be considered [170]. Another ethically tricky option for allotransfer is the potential to use the still-viable mitochondria from a dead human relative in treatment [171, 172]. The final donor source option, xenotransfer, involves the transfer of mitochondria from another species to humans [173]. As unorthodox as it may appear, experiments involving this variation of the AMT did not show apparent mismatch effects in the animal-to-animal models [174]. If xenotransfer of mitochondria between animals and humans were to be successfully executed in vitro or in vivo, numerous ethical concerns would need to be addressed before considering any potential clinical applications.

The use of AMT techniques not involving the transfer of nuclear material raises a number of ethical and safety concerns but may also provide new therapeutic options. One of the key ethical debates related to MST involves the birth of babies with “three parents”; AMT may provide a way to solve this debate by allowing the father's mitochondria and not the donor's to populate the zygote. Another relevant concern in biomedical sciences today deals with the donation of organs and tissues from other people or animals for therapeutic purposes. AMT may allow us to bypass these debates by transferring only microscopic organelles rather than the entire cells or organs to deal and treat certain conditions. The applications of AMT ought to be further explored given these possibilities; however, it is crucial to note that moving from in vitro to in vivo and later to clinical applications AMT implies even greater ethical and biosafety hurdles.

Adequately framing the ethical challenges of cutting edge biomedical procedures like AMT is extremely difficult because these debates are, at least in part, anchored in the sociocultural contexts in which they arise [175]. For example, the UK recently took a major step forward in permitting and regulating the therapeutic applications of MRT by developing a guideline for its use [151]. It should be noted that the UK has been fertile ground for scientific development and that many other countries have not even begun to consider these techniques even for experimentation because of localized ethical and safety concerns [176]. The only way to appropriately address the myriad of ethical and safety issues that impede the global development of this technology is by gathering further evidence on these procedures and facilitating national and international dialogues on these subjects.

## 7. Conclusions

This review analyzes all current AMT techniques and describes the future steps necessary to develop better in vitro, in vivo, and clinical applications. We focus in providing the first academic work known to globally analyze and summarize the field of AMT. To our knowledge, no other publication has compared and contrasted investigators working in in vitro and in vivo applications of AMT. Our review provides a comprehensive summary of the impact of the mitochondria in the cell, the mechanisms through which it is naturally and artificially transferred, and the ethical implications related to its potential clinical applications. This review gives a concise yet detailed overview of the past, present, and future of AMT, which we hope will orient scientists within and outside of this field and to help them contribute to the progress of this technology.

Keeping in mind our goal to orient other scientists, the authors would like to point out three final observations important to the advancement of this field. With respect to the development of AMT techniques, in vitro and in vivo procedures have evolved in a parallel, rather than in a sequential manner [177, 178]. This is unusual in the biomedical field in which techniques are usually first perfected in vitro and then later further developed in in vivo and clinical applications. While this somewhat unorthodox trajectory has certainly produced valuable knowledge, it is also important to recognize that by “skipping steps” scientists may have left important gaps in our knowledge about AMT.

Another potential avenue for scientific investigation not covered in this review is the study of genetic modification of mitochondria before artificially transferring them. Such modifications could range from slight alterations of the mtDNA in order to better facilitate specific cellular processes to the creation of completely artificial “super” mitochondria [179, 180]. As is the case with other genetically modified organisms, this line of investigation not only raises a whole host of ethical, legal, and biosafety questions but also has the potential to greatly benefit humanity if developed correctly.

Finally, the authors would like to critique the terminology used within the field of AMT in service of better elucidating the uniqueness of AMT and its possible therapeutic applications. Investigators currently use the terms mitochondria transformation [9], transfer [93], and transplant [117, 181] interchangeably to designate the artificial transport of mitochondria from one cell to another via diverse methodologies. According to our criteria, the term that best defines this process is *transfer*. Mitochondria can be effectively transferred in vitro and in vivo via a variety of processes such as MitoCeption [10] and Magnetomitotransfer [91]. Mitochondrial “transformation” engenders misunderstandings because the cell is transformed, not the mitochondria. Additionally, because the term “transplant” has come to be associated with tissue and organ transport between donors and recipients, transfer should be used to denote the transport of subcellular components such as mitochondria from one cell to another in order to distinguish the ethical, legal, and biomedical nuances associated with such procedures and to facilitate more exact discussion of these issues.

In order to continue to advance the development of AMT, it is essential that scientists answer questions key to the functioning of these techniques, troubleshoot the challenges for clinical applications, and resolve the ethical, legal, and biosecurity concerns that will determine if this technology is logistically applicable around the world. Addressing these hurdles will enable our generation to unlock the transformative potential of an organelle that was once merely considered the cell power plant.

## Figures and Tables

**Table 1:** A comparative description of MST and PNT techniques and ethical concerns.

	MST	PNT
Technical approach	Transfer of nuclear DNA before fertilization	Transfer of nuclear DNA postfertilization
mtDNA carry-over	Lower (1%)	Higher (1-2%)
Risk of chromosomal abnormalities	Higher	Lower
Operator dependent	Yes	Yes
Proof-of-concept In macaque	Yes (*n* = 4)	No
Ethical issues		
Approval (UK)	Yes	Yes
Approach	Selective reproduction	Therapy based on embryo modification
Manipulation and destruction of	Oocytes	Fertilized eggs
Occurs	Preconception	Postconception

## Abbreviations

AMT: Artificial mitochondria transfer
AIF: Apoptosis-inducing factor
ARDS: Acute respiratory distress syndrome
BAL: Bronchoalveolar lavage
BBB: Blood-brain barrier
BMSCs: Bone marrow-derived stem cells
CAP: Chloramphenicol
CM: Cristae membrane
DRP1: Dynamin-related protein 1
EF: Efrapeptin
EGTA: Ethylene glycol tetraacetic acid
ER: Endoplasmic reticulum
ESCs: Embryonic stem cells
EVs: Extracellular vesicles
FGF-2: Fibroblast growth factor 2
Gfer: Growth factor erv1-like
HEPES: N-(2-hydroxyethyl) piperazine-N′-ethanesulfonic acid
HFEA: Human Fertilization and Embryology Authority
hMSCs: Human mesenchymal stem cells
HPH: Hypoxic pulmonary hypertension
IBM: Inner boundary membrane
IMM: Inner mitochondrial membrane
iPSCs: Induce pluripotent stem cells
LPS: Lipopolysaccharide
MAMs: Mitochondria-associated membranes
MCAO: Middle cerebral artery occlusion
MERRF: Myoclonic epilepsy with ragged red fiber syndrome
Mfn1: Mitofusin 1
Mfn2: Mitofusin 2
MMP-9: Matrix metalloproteinase-9
MRTs: Mitochondria replacements
MSCs: Mesenchymal stem cells
MST: Maternal spindle transfer
mtDNA: Mitochondrial DNA
mTOR: Mammalian target of rapamycin
MVs: Microvesicles
M*β*CD: Methyl-*β*-cyclodextrin
NF-*κ*B: Nuclear factor kappa-light-chain-enhancer of activated B cells
nuDNA: Nuclear DNA
OM: Outer membrane
OMM: Outer mitochondria membrane
OX-PHOS: Oxidative phosphorylation
PEG: Polyethylene glycol
PDM: Parkinson disease model
PINK1: PTEN-induced kinase 1
PMDS: Primary mitochondria diseases
PNT: Pronuclear transfer
RAGE: Receptor for advanced glycation end product
ROS: Reactive oxygen species
TNF *α:*: Tumor necrosis factor alpha
TNTs: Tunneling nanotubes
UV: Ultraviolet
VEGF: Vascular endothelial growth factor.
